# Autoantibodies to selenoprotein P in chronic fatigue syndrome suggest selenium transport impairment and acquired resistance to thyroid hormone

**DOI:** 10.1016/j.redox.2023.102796

**Published:** 2023-07-03

**Authors:** Qian Sun, Elisa Oltra, D.A. Janneke Dijck-Brouwer, Thilo Samson Chillon, Petra Seemann, Sabrina Asaad, Kamil Demircan, José Andrés Espejo-Oltra, Teresa Sánchez-Fito, Eva Martín-Martínez, Waldemar B. Minich, Frits A.J. Muskiet, Lutz Schomburg

**Affiliations:** a Institute for Experimental Endocrinology, Charité-Universitätsmedizin Berlin, Max Rubner Center (MRC) for Cardiovascular Metabolic Renal Research, 10115, Berlin, Germany; b selenOmed GmbH, Berlin, Germany; c Department of Pathology, School of Health Sciences, Universidad Católica de Valencia San Vicente Mártir, Valencia, Spain; d Department of Laboratory Medicine, University Medical Center Groningen, University of Groningen, Hanzeplein 1, 9713 GZ, Groningen, the Netherlands; e National Health Service, Manises Hospital, Valencia, Spain

**Keywords:** SELENOP, Trace element, Redox, COVID, Autoimmunity, Glutathione peroxidase

## Abstract

Chronic Fatigue Syndrome (CFS) presents with symptoms of hypothyroidism, including mental and physical fatigue, poor sleep, depression, and anxiety. However, thyroid hormone (TH) profiles of elevated thyrotropin and low thyroxine (T4) are not consistently observed. Recently, autoantibodies to the Se transporter SELENOP (SELENOP-aAb) have been identified in Hashimoto's thyroiditis and shown to impair selenoprotein expression. We hypothesized that SELENOP-aAb are prevalent in CFS, and associate with reduced selenoprotein expression and impaired TH deiodination.

Se status and SELENOP-aAb prevalence was compared by combining European CFS patients (n = 167) and healthy controls (n = 545) from different sources. The biomarkers total Se, glutathione peroxidase (GPx3) and SELENOP showed linear correlations across the samples without reaching saturation, indicative of Se deficiency. SELENOP-aAb prevalence was 9.6–15.6% in CFS versus 0.9–2.0% in controls, depending on cut-off for positivity. The linear correlation between Se and GPx3 activity was absent in SELENOP-aAb positive patients, suggesting impaired Se supply of kidney. A subgroup of paired control (n = 119) and CSF (n = 111) patients had been characterized for TH and biochemical parameters before. Within this subgroup, SELENOP-aAb positive patients displayed particularly low deiodinase activity (SPINA-GD index), free T3 levels, total T3 to total T4 (TT3/TT4) and free T3 to free T4 (FT3/FT4) ratios. In 24 h urine, iodine concentrations were significantly lower in SELENOP-aAb positive than in SELENOP-aAb negative patients or controls (median (IQR); 43.2 (16.0) vs. 58.9 (45.2) vs. 89.0 (54.9) μg/L). The data indicate that SELENOP-aAb associate with low deiodination rate and reduced activation of TH to active T3.

We conclude that a subset of CFS patients express SELENOP-aAb that disturb Se transport and reduce selenoprotein expression in target tissues. Hereby, TH activation decreases as an acquired condition not reflected by thyrotropin and T4 in blood. This hypothesis opens new diagnostic and therapeutic options for SELENOP-aAb positive CFS, but requires clinical evidence from intervention trials.

## Highlights

• Comprehensive analysis of Se and thyroid hormone in chronic fatigue syndrome.
• Biomarkers of Se status, i.e., total Se, GPx3 and SELENOP correlate linearly.
• Autoantibodies to SELENOP are relatively frequent in chronic fatigue syndrome.
• SELENOP-aAb are associated with dysregulated GPx3 and DIO expression.
• Strongly reduced urinary iodine reflects low TH activation and DIO activity.

## Graphical abstract

**Figure:**
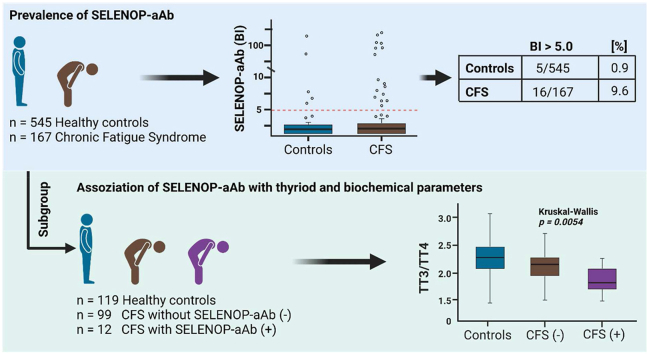

## 1 Introduction

Thyroid hormone (TH) is a central regulator of temperature, energy metabolism and psychic well-being. Feedback control of the TH axis is mainly elicited via suppressive effects of circulating TH on thyrotropin-releasing hormone (TRH) from hypothalamic neurons and thyrotropin (TSH) from endocrine cells of the anterior pituitary [1]. The majority of circulating TH consists of the prohormone thyroxine (3,3′,5,5′-tetraiodo-L-thyronin, T4) which is converted in target cells by deiodination to the active TH derivative tri-iodothyronine (3,3′,5-triiodo-L-thyronin, T3). The cleavage of the C–I bond is catalyzed by one of three selenium (Se)-dependent deiodinase isozymes (DIO) [2,3]. DIO1 and DIO2 are capable of converting T4 to active T3, whereas DIO3 converts T4 to inactive reverse-T3 (rT3), and T3 to T2, respectively. All three DIO isozymes are selenoproteins, thereby relying on regular Se supply for biosynthesis [4]. Direct interactions between Se status and TH metabolism are described, and may involve altered DIO expression due to inflammation, single nucleotide polymorphisms, a low nutritional supply of the trace element, or other modifiers [[5], [6], [7]]. A direct interaction between TH metabolism and Se status has been identified in regions with endemic micronutrient deficiency, where correcting the Se deficit increased TH degradation and iodine loss, thereby aggravating disease symptoms in myxoedematous cretinism [8]. Hence, Se deficiency may reduce TH metabolism and affect iodine excretion.

A tumor-associated overexpression of DIO3 can lead to consumptive hypothyroidism due to increased TH inactivation, further supporting the central role of deiodination in TH status control [9]. Conversely, impaired deiodination of mono- and di-iodotyrosine residues due to inherited mutations in dehalogenase 1 (*DEHAL1*) causes developmental retardations, urinary iodine loss and congenital hypothyroidism [10].

Patients with chronic fatigue syndrome (CFS) display several symptoms compatible with hypothyroidism, in particular physical and mental fatigue, depression, fibromyalgia (FM) and hypometabolism with low energy stores [[11], [12], [13]]. Accordingly, TH patterns in blood of patients with CFS have been analyzed and certain alterations with some resemblance to the clinical picture of critical illness with characteristically elevated rT3 and low T3 concentrations are reported, and described as a mild form of low-T3 syndrome [11,14,15]. In the case of FM, an association between FM diagnosis and thyroid autoimmunity has driven clinical trials with TH supplementation, albeit without clear general benefits [16,17]. The TH alterations observed in CFS could result from impaired DIO expression; however, due to their nature as intracellular enzymes, DIO activities cannot easily be assessed in human subjects as the target tissues are not readily accessible for analysis [2,13,18].

A common cause of hypothyroidism is Hashimoto's thyroiditis (HT), where infiltrating lymphocytes destroy the follicular thyroid structure, eventually leading to chronic hypothyroidism requiring T4 substitution [19]. However, a significant fraction of patients on T4 monotherapy report reduced quality of life [20]. We have recently identified natural autoantibodies (aAb) to the Se transporter selenoprotein P (SELENOP) in a subgroup of patients with HT [21]. The physiological consequence of SELENOP-aAb included an impaired Se transport into target cells causing intracellular Se deficiency *in vitro* [21,22]. This notion was supported by particularly low activity of kidney-derived plasma glutathione peroxidase (GPx3), as its biosynthesis depends on liver-derived SELENOP [23,24]. Hence, SELENOP-aAb may cause intracellular Se deficiency in SELENOP-target cells of the endocrine, central nervous and immune system [25]. In the present study, we tested the hypothesis that SELENOP-aAb are prevalent in patients with CFS, potentially causing impaired DIO expression and hypothyroidism.

## 2 Materials and methods

### 2.1 Human samples and associated data

All procedures were conducted in agreement with the ethical standards as laid down in the Helsinki Declaration, revised in 2008, and all the patients included had given their written informed consent. The study protocols were approved by the local authorities at the University Medical Center Groningen (UMCG), (Ethical committee, NL44299.042.13, METc 2013/154), and the Public Health Research Ethics Committee DGSP-CSISP, Valencia (núm. 20190301/12 and 20210604/04/01). For the analysis of Se status and prevalence of SELENOP-aAb, three control groups were combined, i.e., samples from apparently healthy subjects recruited in Groningen, The Netherlands (n = 119) [11], and Valencia, Spain (n = 26) [26], combined with subjects giving a self-reported assessment of “healthy” (n = 400) from a commercial supplier near Berlin, Germany (InVent Diagnostica GmbH, Hennigsdorf, Germany) [21]. The composite CFS group consisted of two sets of patients, complying with the Canadian and/or international case definitions, recruited from the Parkstad Clinic in Amsterdam, The Netherlands (n = 111), and in Valencia, Spain (n = 56).

The comparison of parameters reflecting the TH axis and of biochemical metabolites was conducted with the subjects from Groningen, The Netherlands, as these patients and controls represented matched samples, with similar anthropometric characteristics including gender distribution, age, height, weight and BMI (Supplementary Table 1), as reported recently [11]. Moreover, the TH and biochemical parameters of the samples had been determined before according to highest quality standards by experienced analytical laboratory methods [11].

### 2.2 Assessment of biomarkers of Se status

In order to yield a comprehensive assessment of Se status, four biomarkers were determined, namely total serum/plasma Se and SELENOP concentrations, enzymatic activity of GPx3 and SELENOP-aAb titers, as described [21,27]. Briefly, total Se was determined by total reflection X-ray fluorescence (TXRF) analysis from diluted samples spiked with a gallium supplement for standardization. Precision as well as intra- and inter-assay variations were determined with a commercial standard serum (Seronorm, SERO, Billingstad, Norway), and in-house standards with known Se concentrations, yielding variations of <10%, as described [27,28]. SELENOP was determined by a validated sandwich ELISA (selenOtest ELISA, selenOmed GmbH, Berlin, Germany), yielding coefficients of variation <10% for the provided control samples with low or medium SELENOP concentrations, as described [[27], [28], [29]]. The enzymatic activity of GPx3 was assessed in triplicates by an enzymatic test using hydroperoxide as acceptor substrate, as originally described by Flohé and Güntzler [30], using a constant pool standard serum for quality control, yielding variation coefficients of <15% during the analyses, as described [28]. The SELENOP-aAb were measured by a fusion protein consisting of human SELENOP coupled in frame to secreted embryonic alkaline phosphatase (SEAP) as reporter. Immune-complexes were precipitated by protein A, washed extensively and quantified by luminometric analysis of SEAP activity, as described [21]. A negative and two SELENOP-aAb positive human serum samples were used as standards during the measurements, as described [21]. SELENOP-aAb titers are expressed as binding index (BI), denoting the signal strength as factor above background control signals [31].

### 2.3 Analysis of biomarkers of oxidative stress and thyroid hormone status

The analysis of biomarkers of oxidative stress and serum concentrations of TSH, free T4 (FT4), free T3 (FT3), total T4 (TT4) and total T3 (TT3) in the control and CFS samples from Groningen, The Netherlands, was conducted prior to this study by participating laboratories in Amsterdam and Delft, using routine hematological analyzers, LC-electrospray ionization-MS/MS, and commercial immunoassays on laboratory automats from Roche and Abbott, as described [11]. The concentrations of rT3 were determined by an in-house RIA at the Amsterdam Medical Center, as described [11]. The indices SPINA GT and SPINA GD as biomarkers of the secretory capacity of the thyroid gland (SPINA-GT) and DIO activity (SPINA-GD), respectively, were calculated and correlated to SELENOP-aAb [11,32,33]. Urinary iodine was determined from 24-h urine samples, as described [11]. F2-isoprostane concentrations were determined by GC-tandem-MS after derivatization and solid-phase extraction, as described [11,34].

### 2.4 Western blot analysis

A preselected set of sample aliquots was subjected to Western blot analysis using a monoclonal antibody to human SELENOP (1:2.000 dilution, SM-MAB-7635, selenOmed). To this end, serum was diluted 1:20, supplemented with reducing buffer, and 20 μl (equivalent to 0.74 μl serum) were separated in 12.5% sodium dodecyl sulfate - polyacrylamide gel electrophoresis (SDS-PAGE). The gel was blotted by semi-dry transfer onto a nitrocellulose membrane (0.45 μm, Protran, GE Healthcare, Uppsala, Sweden). Detection was achieved by using anti-mouse IgG-HRP (1:2.500 dilution, NXA931, GE Healthcare GmbH, Solingen, Germany), and signals were recorded by X-ray film (Amersham), as described [35]. Quantification of signal strengths was done by ImageJ analysis of band intensities detected by the X-ray film (NIH, Bethesda, USA).

### 2.5 Statistical analyses

Statistical analyses were conducted using GraphPad Prism v.9.1.2 (GraphPad Software Inc., San Diego, USA), or the R software, version 4.1.1, implementing the packages dplyr, tidyr, gtsummary and ggplot2. Power calculation was conducted with G*Power, version 3.1.9.7 [36]. The required group size of at least n = 146 CSF patients was calculated, based on the prevalence of SELENOP-aAb in thyroid disease and cancer [21,22], expecting 10% of positive CFS patients, an alpha level set at 0.05, and a power (1-beta) of 0.8, considering the variation observed for SPINA-GD in CFS versus control (13.4 vs. 15.7, SD 2.5) as readout of DIO activity [11]. The results are represented as mean with SD, as median with interquartile range, or by displaying the individual values. Number of missing values are indicated. Correlation of Se biomarkers was assessed using Spearman's rank correlation test, and prevalence of SELENOP-aAb positivity was determined in relation to the binding index (BI), denoting the signal strength over background, as described [21,22]. The comparison of TH and biochemical parameters was conducted by the Kruskal-Wallis test, analyzing the matched samples of controls and CSF patients from Groningen (n = 119 and n = 111, respectively), subdivided into SELENOP-aAb positive versus negative subjects. *P*-values <0.05 were considered significant and are indicated in the tables and figures. As the thyroid-related parameters are interrelated, and the character of this study is an explorative analysis, no correction for multiple testing was applied.

## 3 Results

### 3.1 Prevalence of SELENOP-aAb in patients and controls

**Fig. 1:**
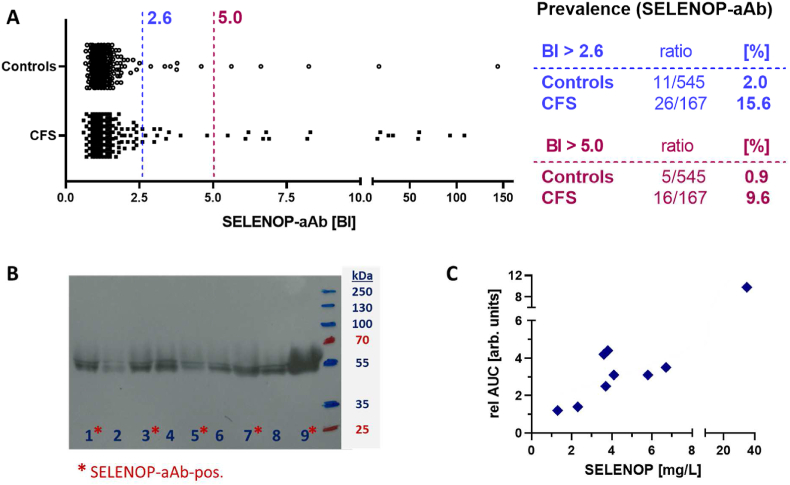
Prevalence of SELENOP-aAb in patients and controls, along with Western blot and ELISA analysis of SELENOP. (A) All control (Controls) and CFS patient (CFS) samples were analyzed for autoimmunity to the Se transporter SELENOP (SELENOP-aAb).. The concentration of SELENOP-aAb is expressed as binding index (BI), indicating the signal strength above background noise from negative controls. The broken lines indicates the cut-offs used to classify positive signals, calculated from the central 95% of controls by adding three times the standard deviation to the mean (BI_threshold_ = Avrg+3SD => 1.197 + 3*0.465 = 2.591, blue), or using BI = 5.0 (magenta) as a stringent cut-off. The prevalence of SELENOP-aAb is indicated as ratio and percentage in magenta (BI > 5.0) and blue (BI > 2.6), respectively. (B) Selected samples with or without SELENOP-aAb were subjected to Western blot analysis (SELENOP-aAb-positive: #1, 3, 5, 7, 9). Migration pattern was independent of SELENOP-aAb. (C) Semi-quantitative analysis of band strength was compared to the ELISA data and indicated a linear positive correlation. The results verify that SELENOP-aAb did not interfere with ELISA quantification; x-axis; ELISA result (mg/L), y-axis; Western blot analysis. (For interpretation of the references to colour in this figure legend, the reader is referred to the Web version of this article.)

The full sample collection was used to compare the prevalence of SELENOP-aAb in controls and CFS, which consisted of 545 controls and 167 patients with CFS, collected in Groningen, The Netherlands, and Valencia, Spain, supplemented by additional controls from a commercial supplier in Berlin, Germany. The cut-off for positivity was determined from the control samples, and calculated as mean plus three times standard deviation of the central 95% of readings, comprising theoretically 99.7% of normally distributed samples (Fig. 1A). Hereby, a value of BI = 2.6 resulted as threshold for separating SELENOP-aAb negative from SELENOP-aAb positive samples. As prior studies indicated dose-dependent effects of SELENOP-aAb, a second more stringent threshold of BI = 5.0 was applied to specifically analyze highly positive subjects only, as done earlier [21,22]. According to these thresholds, prevalence of SELENOP-aAb was about ten times higher in CFS than in controls (BI > 2.6; 15.6% vs. 2.0%, or BI > 5.0; 9.6% vs. 0.9%) (Fig. 1A). Western blot analyses revealed a similar band pattern across all samples, irrespective of the presence of SELENOP-aAb (Fig. 1B). Semi-quantitative assessment of the Western blot results correlated linearly to the ELISA-based quantification, and indicated no interfering effects of natural SELENOP-aAb on the immunochemical analysis of SELENOP concentrations (Fig. 1C).

### 3.2 Correlation analysis of Se biomarkers in samples with or without SELENOP-aAb

**Fig. 2:**
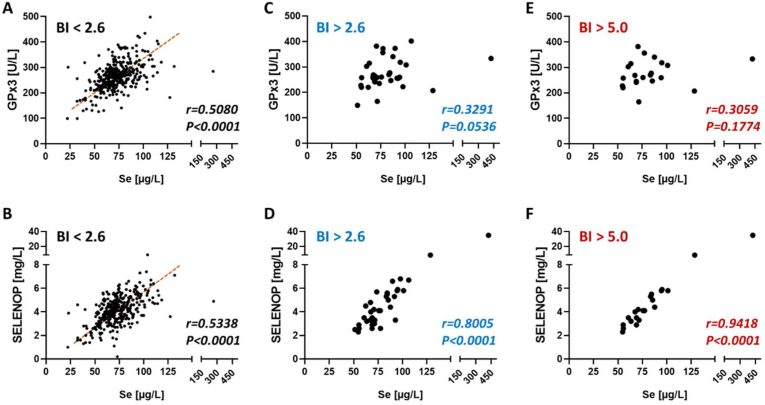
Correlation analysis of biomarkers of Se status in relation to autoimmunity to the Se transporter. All samples available from the clinical cohorts were analyzed for SELENOP-aAb, and classified according to the binding index (BI; factor over background control signal) as positive or negative. According to a mathematical outlier criterion, a BI of 2.6 was used as threshold in the first analysis (A-D), and a more stringent BI of 5.0 to test highly positive samples only was used in the second analysis (E, F). (A) In negative samples, Se concentration and GPx3 activity showed a strong linear correlation, indicating that the groups are Se deficient as the expression of GPx3 shows no saturation. (B) Similarly, Se and SELENOP were linearly correlated, again indicative of Se deficiency. (C) In the group of SELENOP-aAb positive samples (BI > 2.6), no significant correlation of total Se and GPx3 activity was observed, whereas (D) a strong positive correlation of Se and SELENOP was given. (E) In highly positive samples (SELENOP-aAb; BI > 5), no correlation of Se and GPx3 was detected. (F) However, Se and SELENOP correlated stringently and positively in the samples displaying high titers of SELENOP-aAb, reflecting the notion that SELENOP contributes decisively to total Se concentrations. Analyses of Se biomarkers by non-parametric Spearman correlation. r; Spearman's rank correlation coefficient, *P;* significance level.

All the samples collected in the clinics in Groningen and Valencia were analyzed for total Se and SELENOP, GPx3 activity and SELENOP-aAb. Using SELENOP expression saturation as threshold for replete Se status, i.e., serum or plasma Se > 125 μg/L, the results indicate Se deficiency for almost all samples (Fig. 2). This notion is supported by the positive linear correlation of SELENOP and total Se concentration. Severe deficiency with Se concentrations <45.7 μg/L were observed in one of the control samples (44.6 μg/L) from The Netherlands, and none of the Spanish control samples, but in ten of the Dutch CFS patients (9%) and in one of the Spanish CFS patients (2%). Notably, none of the subjects with severe Se deficiency was positive for SELENOP-aAb. The samples with BI < 2.6 were classified as SELENOP-aAb negative and displayed the expected linear correlation of Se and GPx3 activity (Fig. 2A). Similarly, Se and SELENOP showed a positive linear correlation in the SELENOP-aAb negative samples (Fig. 2B). In the group of SELENOP-aAb positive samples (BI > 2.6), the same analysis was conducted and yielded no correlation between Se and GPx3 activity (Fig. 2C). At the same time, SELENOP and Se showed a linear correlation, supporting the notion that SELENOP represents the major selenoprotein in blood, irrespective of SELENOP-aAb (Fig. 2D). Raising the threshold for SELENOP-aAb to a stringent cut-off of BI > 5.0, the same interrelationships were observed, with a lack of correlation between Se and GPx3 activity (Fig. 2E), but a strong linear correlation between Se and SELENOP (Fig. 2F).

### 3.3 Biomarkers of inflammation and oxidative stress in relation to SELENOP-aAb

**Fig. 3:**
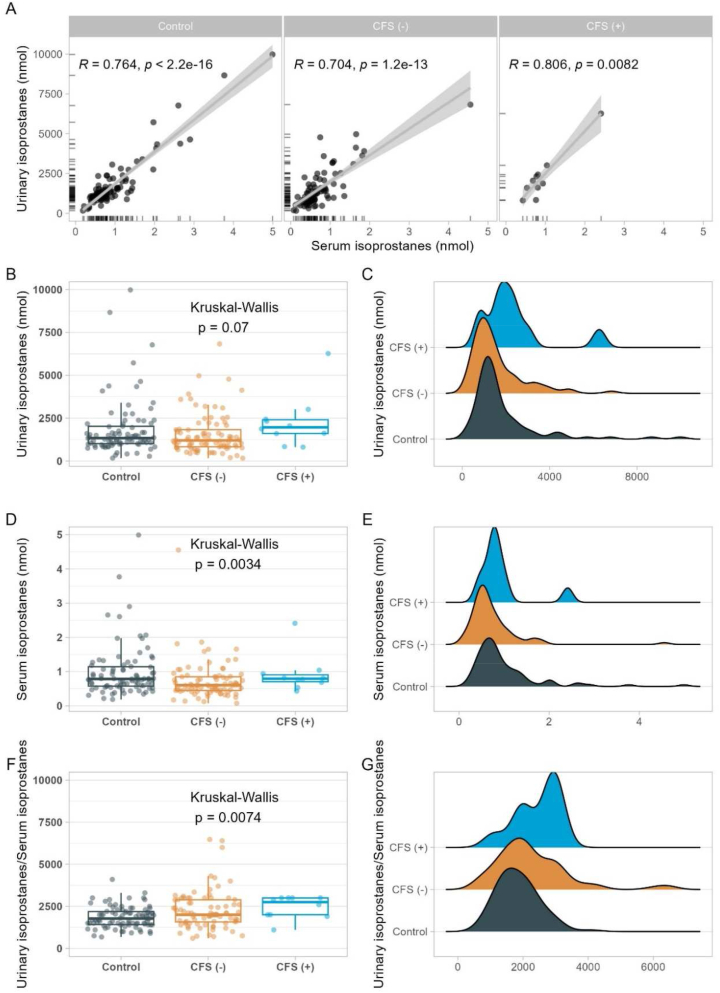
Isoprostane concentrations in relation to SELENOP-aAb. (A) The samples from control and CFS patient (CFS(+); BI (SELENOP-aAb) >5.0) showed linear positive correlations of urinary and serum isoprostanes. (B, C**)** Urinary isoprostanes were relatively high in SELENOP-aAb positive samples, but did not differ when all three groups were compared. (D, E) Serum isoprostanes were relatively low in SELENOP-aAb negative CFS patients. (F) The ratio of urinary over serum isoprostanes was significantly elevated in the group of SELENOP-aAb positive CFS patients, with (G**)** the majority of data shifted to the right in the density plot. Interrelations analyzed by Spearman rank correlation, and groups compared by Kruskal-Wallis test (results as insets).

The samples from controls (n = 119) and CSF patients (n = 111) recruited in Groningen, The Netherlands, had been intensively characterized with respect to biomarkers of inflammation and oxidative stress in a prior analysis [11]. Within this cohort, 12 CFS patients displayed strongly positive SELENOP-aAb (BI > 5). Comparing the SELENOP-aAb positive with the SELENOP-aAb negative samples, there was no significant difference in age, sex ratio or BMI (Supplementary Table 1). In previous analyses, SELENOP-aAb have been associated with impaired Se transport and reduced selenoprotein expression [21,22], potentially causing oxidative stress. In a direct comparison of the samples from controls versus SELENOP-aAb positive and negative CFS patients, several of the tested biomarkers showed no significant differences between the groups, namely the inflammation biomarkers hsCRP, white blood cell count (WBC) and the tryptophan/kynurenine ratio (Table 1). Serum and urinary isoprostane concentrations presented an inhomogeneous picture. A direct comparison indicated that both biomarkers of oxidative stress status showed a linear positive correlation in all three groups of samples (Fig. 3A). The detailed analysis indicated that urinary isoprostanes are elevated in the presence of SELENOP-aAb (Fig. 3B), and a particular shift to higher concentrations was identified for a particular subgroup in the density plot (Fig. 3C). Serum isoprostanes were particularly low in SELENOP-aAb negative patients (Fig. 3D, E). The direct comparison of the isoprostanes in both matrices indicated a trend of the ratio of urinary over serum isoprostanes, increasing from controls via SELENOP-aAb negative to SELENOP-aAb positive CFS patients (Fig. 3F), again with an apparent characteristic right shift of a subgroup of SELENOP-aAb positive CFS patients in the density plot (Fig. 3G).

**Table 1:** Biomarkers of oxidative stress in relation to SELENOP-aAb.

Variable	Control	CFS patients		
	n = 119^a^	SELENOP aAb (−) n = 99^a^	SELENOP aAb (+) n = 12^a^	p-value^b^
**hsCRP**	0.77 (0.90)	0.91 (1.69)	1.24 (1.66)	0.46
(Missing)	20	12	2	
**WBC**	6.30 (2.19)	6.10 (1.95)	5.25 (1.93)	0.22
(Missing)	20	12	2	
**Kynurenine**	1.81 (0.49)	1.62 (0.61)	1.59 (0.76)	**0.004**
(Missing)	20	12	2	
**Tryptophan**	56.40 (11.90)	54.00 (15.25)	56.35 (13.27)	**0.012**
(Missing)	20	12	2	
**Tryptophan/Kynurenine**	32.42 (9.38)	32.70 (9.95)	31.37 (10.19)	0.53
(Missing)	20	12	2	
**Urinary isoprostanes**	1336.35 (1011.60)	1199.57 (998.78)	1963.00 (803.75)	0.070
(Missing)	33	15	2	
**Serum Isoprostanen**	0.78 (0.57)	0.60 (0.40)	0.79 (0.20)	**0.003**
(Missing)	33	16	2	
**Urinary isoprostanes/Serum Isoprostanen**	1775.00 (775.00)	2000.00 (1315.00)	2750.00 (1000.00)	**0.007**
(Missing)	33	16	2	
**DNL liver**	36.26 (2.22)	34.98 (1.95)	35.05 (2.64)	**<0.001**
(Missing)	20	12	2	
**Zonulin**	1.39 (0.49)	1.24 (0.46)	1.32 (0.33)	**0.011**
(Missing)	20	12	2	

a Median (IQR).

b Kruskal-Wallis rank sum test.

### 3.4 Association of SELENOP-aAb with parameters of the thyroid hormone axis

Besides the characterization of biomarkers of inflammation and oxidative stress, all the samples from Groningen, The Netherlands, had undergone a comprehensive analysis of the TH profiles by standardized technology [11]. The analyses included thyrotropin (TSH), free and total T4 and T3 concentrations, rT3 levels, the percentages of free fraction of T4, T3 and rT3 (%TT4, %TT3, %rT3), the different TH ratios along with SPINA GT and SPINA GD indices, and urinary iodine concentrations. An exploratory analysis was performed with the full set of these parameters to analyze for a potential disruption of TH metabolism by SELENOP-aAb. Several significant differences between patients divided into those with or without SELENOP-aAb were observed in comparison to the controls, and a complete overview is provided (Table 2). The concentrations of TSH, FT3 and rT3 showed no significant differences across the three groups. The SELENOP-aAb positive CFS patients displayed particularly elevated FT4 and rT3/TT4 as well as rT3/TT3 concentrations. The majority of other indices of the TH axis appeared suppressed in CFS, with lowest values in the SELENOP-aAb positive patients. This particular characteristic applies to the percentage of free fraction of T3 (%TT3), and the ratio of TT3/TT4, FT3/FT4, and TT3/FT3, collectively indicating a reduced rate of TH activation (T4 to T3 conversion), as observed before in patients with inherited defects in selenoprotein expression [37].

**Table 2:** Comparison of indices of thyroid hormones and deiodination.

Variable	Control	CFS patients		
	Control	SELENOP aAb (−)	SELENOP aAb (+)	p-value^b^
	n = 119^a^	n = 99^a^	n = 12^a^	
**TSH (mU/L)**	1.59 (1.04)	1.44 (0.81)	1.25 (0.80)	0.44
(Missing)	21	12	2	
**FT4(pmol/L)**	15.6 (2.7)	15.8 (3.2)	17.9 (1.9)	**0.005**
(Missing)	20	12	2	
**FT3 (pmol/L)**	5.20 (1.25)	5.20 (1.10)	4.85 (1.25)	0.12
(Missing)	20	12	2	
**TT4 (nmol/L)**	72.0 (15.3)	62.8 (27.6)	64.1 (17.1)	**<0.001**
(Missing)	20	12	2	
**TT3 (nmol/L)**	1.60 (0.40)	1.40 (0.68)	1.45 (0.50)	**<0.001**
(Missing)	20	13	2	
**rT3 (nmol/L)**	0.23 (0.07)	0.22 (0.08)	0.28 (0.11)	0.25
(Missing)	20	12	2	
**% TT4**	97.6 (0.4)	97.5 (0.4)	97.9 (0.2)	**0.033**
(Missing)	20	13	2	
**% TT3**	2.14 (0.44)	2.07 (0.40)	1.80 (0.38)	**0.005**
(Missing)	20	13	2	
**% rT3**	0.30 (0.09)	0.34 (0.15)	0.35 (0.12)	**<0.001**
(Missing)	20	13	2	
**TT3/TT4**	2.19 (0.46)	2.12 (0.42)	1.84 (0.39)	**0.005**
(Missing)	20	13	2	
**FT3/FT4**	34.0 (8.5)	32.6 (5.6)	27.7 (3.7)	**<0.001**
(Missing)	20	12	2	
**rT3/TT4**	0.31 (0.09)	0.34 (0.15)	0.35 (0.12)	**<0.001**
(Missing)	20	12	2	
**rT3/TT3**	1.47 (0.57)	1.76 (0.94)	1.86 (1.18)	**<0.001**
(Missing)	20	13	2	
**TT3/FT3**	0.31 (0.05)	0.28 (0.12)	0.25 (0.08)	**0.002**
(Missing)	20	13	2	
**TT4/FT4**	46.3 (10.4)	40.8 (16.9)	37.9 (12.6)	**<0.001**
(Missing)	20	12	2	
**SPINA GT (pmol/s)**	2.08 (1.01)	1.65 (1.14)	2.04 (0.84)	**0.007**
(Missing)	21	12	2	
**SPINA GD (nmol/s)**	15.8 (4.5)	13.6 (6.2)	13.1 (5.5)	**<0.001**
(Missing)	20	13	2	
**Iodine urine (μg/d)**	89.0 (54.9)	58.9 (45.2)	43.2 (16.0)	**<0.001**
(Missing)	32	15	2	
**SPINA GD x Iodine urine**	1329.0 (1006.7)	665.3 (675.6)	481.8 (258.4)	**<0.001**
(Missing)	32	16	2	

a Median (IQR).

b Kruskal-Wallis rank sum test.

In addition to TH concentrations, two calculated indices of the thyroid gland activity and the rate of peripheral deiodination, respectively, were determined and compared, namely the SPINA GT index, describing the calculated secretory capacity of the thyroid gland, and the SPINA GD index, i.e., the calculated total step-up DIO activity. The SELENOP-aAb positive patients displayed lowest SPINA GD values, compatible with the former findings on reduced TH activation. This notion is further supported when comparing 24 h urinary iodine concentrations, where control subjects showed on average two-times higher values than CFS patients (Median (IQR); 89.0 (54.9) vs. 43.2 (16.0) μg/L, P < 0.001), indicative of suppressed TH deiodination in CFS (Table 2).

**Fig. 4:**
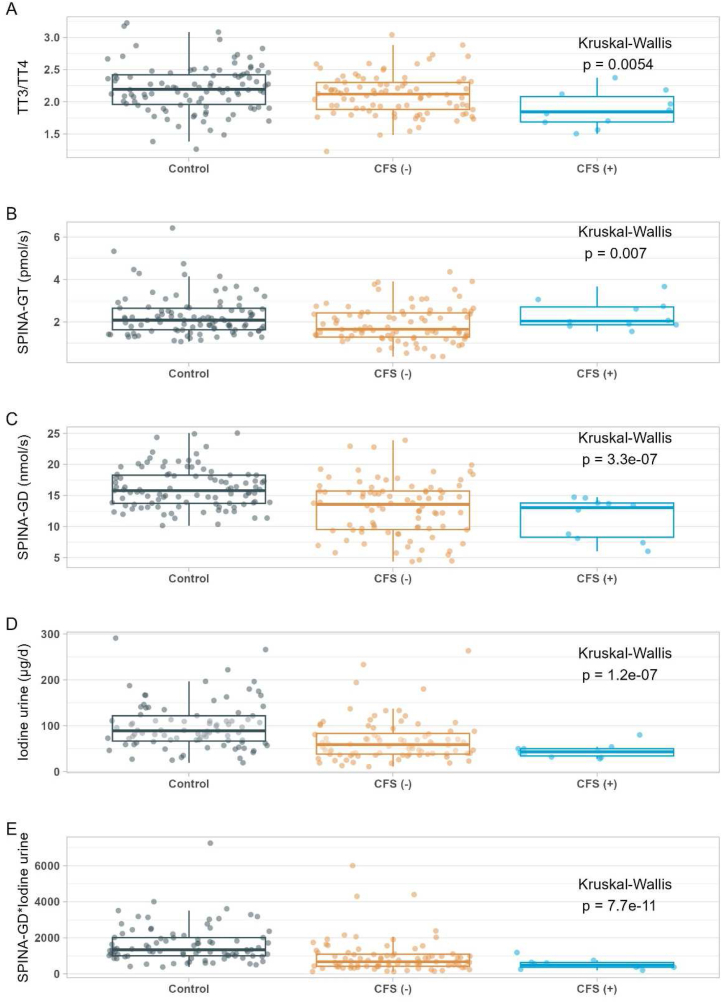
Indices of thyroid hormone metabolism and urinary iodine in controls versus CFS patients divided into those with or without SELENOP-aAb. The samples from the CFS patients are separated according to autoimmunity, with CFS(−) indicating samples with a BI < 5.0, and CFS(+) indicating samples with a BI > 5.0 with regards to SELENOP-aAb. (**A)** The ratio of total hormone concentrations of T4 (TT4) and T3 (TT3) differed between the groups. (B) The calculated secretory capacity of the thyroid gland (SPINA-GT) was relatively low in the group of CFS patients, but not different between controls and CFS patients with SELENOP-aAb. (C) The sum activity of peripheral deiodinases (SPINA-GD) was lowest in CFS patients with SELENOP-aAb. (D) In line with reduced deiodinase activity, concentration of urinary iodine was particularly low in SELENOP-aAb positive CFS patients. (E) The product of SPINA GD and urinary iodine emerged as a most sensitive index characterizing the SELENOP-aAb positive CFS patients. Comparisons conducted by the Kruskal-Wallis test, results indicated as insets.

In order to provide a detailed overview on some of the most instructive findings, a set of direct comparisons between controls versus the two CFS groups separated by SELENOP-aAb presence is presented as graphics displaying all individual data points (Fig. 4). The ratio of TT3/TT4 appeared to decline from control to CFS patients and further to SELENOP-aAb positive CFS patients (Fig. 4A). The SPINA GT index, i.e., the calculated secretory capacity of the thyroid gland, was relatively low in CFS, but not in the SELENOP-aAb positive CFS patients as compared to controls (Fig. 4B). In contrast, the SPINA GD index, i.e., the calculated total step-up DIO activity, was particularly low in the SELENOP-aAb positive CFS patients (Fig. 4C). In line with low SPINA GD and reduced deiodination, SELENOP-aAb positive CFS patients displayed particularly low urinary iodine, notably in a narrow concentration range (Fig. 4D). While the controls presented a median urinary iodine in the range of 100 μg/L, at the border of insufficient to adequate supply, all the SELENOP-aAb positive CFS patients would be classified as iodine insufficient. Accordingly, among all the potential parameters tested, the product of SPINA GD and urinary iodine seems to constitute the most sensitive and informative index to identify CFS patients with reduced TH metabolism and low deiodination rate (Fig. 4E).

## 4 Discussion

This study describes autoimmunity to Se transport in a considerable subset of patients with CFS. The identified SELENOP-aAb were associated with a dysregulation of GPx3 expression and DIO activity, elevated isoprostane concentrations, reduced TH deiodination and low urinary iodine excretion. The prevalence of SELENOP-aAb in CFS was about 10-times elevated in comparison to controls, and exceeded the prevalence reported before in thyroid or cancer patients [21,22]. The findings support the notion that SELENOP-aAb impair regular Se supply to target tissues and local selenoprotein expression, as reflected in the absent correlation of Se or SELENOP levels with kidney-derived GPx3 activity in blood. Reduced renal GPx3 expression in association with elevated urinary isoprostanes argues for Se deficiency in kidney. This notion is supported by the TH parameters measured in SELENOP-aAb positive CFS patients, namely a reduced SPINA 10.13039/100004690GD index in combination with a low T3 to T4 ratio. The low urinary iodine observed in CFS patients with SELENOP-aAb strengthens the interpretation of target cell Se deficiency, as it points to a systemically reduced DIO expression and low deiodination rate, causing little liberation of iodide from TH. This notion is compatible to the effects observed in *Dio1*-knockout mice, where urinary iodine was similarly depressed, as it shifted from urine to faeces in the form of non-deiodinated iodothyronines [38].

In contrast to these differences, no consistent effects of SELENOP-aAb on serum biomarkers of metabolic stress or inflammation were observed. This finding is in agreement with the notion that the majority of patients displayed a similar Se status as the controls, irrespective of the presence of SELENOP-aAb [21,22]. The measured indices of general inflammation, hsCRP and WBC, were not different between the patients positive or not for SELENOP-aAb, but showed high inter-individual variability, which hampers interpretation and indicates high heterogeneity within the patient group. Similarly, no differences were observed in the circulating parameters of metabolic health. This finding may relate to the nature of SELENOP-aAb, acting downstream of liver and disrupting Se transport to target tissues, without causing Se deficiency in hepatocytes [39]. Besides these metabolic and inflammation makers, isoprostanes are particularly informative, and have been established as most reliable biomarkers of oxidative stress and lipid peroxidation rate in clinical studies [40,41]. Isoprostanes are a relatively stable parameter, in particular in urine where lipid concentration is low. They reflect radical-catalyzed oxidation of arachidonic acid independent of enzymatic cyclooxygenase activity [42]. Elevated urine levels were reported in response to stress and disease, such as cigarette smoking, in atherosclerosis, neurodegeneration, or age-related macular degeneration [43,44].

Under normal conditions, there is linear correlation between serum and urinary isoprostane concentrations, as in our study, unless there are dynamic processes like acute stress, infections or elevated renal oxidation leading to particularly high urinary levels [43,45]. Nutrition, and in particular Se status and selenoproteins have been shown to affect urinary isoprostane concentrations *in vivo*. Direct interactions were proven in a porcine model after feeding thermally peroxidized soybean oil, where Se-dependent GPx activity decreased and urinary isoprostanes were elevated [46]. In adult human subjects, high Se status at baseline was predictive for relatively low urinary isoprostanes levels [47]. Importantly, a correlation to metabolic or inflammatory markers in blood was not observed under these conditions, such as hsCRP or IL-6 [47], further supporting the suitability of isoprostanes as valuable readout for assessing oxidative stress in relation to Se status and selenoprotein expression.

**Fig. 5:**
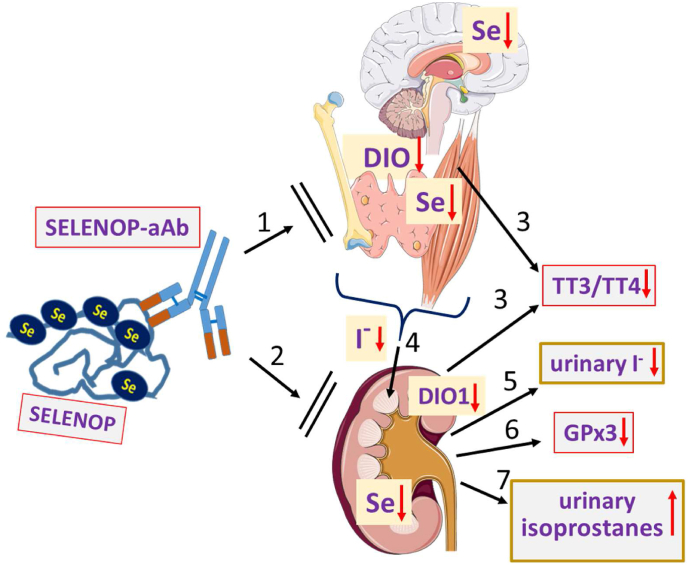
Overview on the data and postulated pathways affected by SELENOP-aAb in CFS. Autoantibodies to SELENOP (SELENOP-aAb) impair Se transport to (1) Se-dependent tissues expressing SELENOP-receptors, including (2) the kidney. Consequently, tissue Se status is low, with suboptimal deiodinase (DIO) expression, which is reflected in a low DIO activity index (SPINA GD), and (3) a suppressed TT3 to TT4 ratio in blood. DIO deficiency also causes (4) reduced iodide (I^−^) liberation from iodothyronines, and (5) low urinary iodine excretion. The low levels of (6) extracellular GPx3 activity indicate renal Se deficiency with high oxidative stress, reflected in (7) elevated urinary isoprostane concentrations. The model above is based on the analyses of serum and urine biomarkers (grey background with framed boxes); the postulated changes in the tissues (center; yellow background) are compatible with the data, but were theoretically deduced as direct tissue biosamples from human subjects were unavailable. (For interpretation of the references to colour in this figure legend, the reader is referred to the Web version of this article.)

The postulated defects in Se-dependent DIO activity were also not readily reflected in blood by routine biomarkers of the TH axis, i.e. total T4 and TSH, as there was no difference between patients and controls, or with respect to SELENOP-aAb. This notion accords with the characterization of subjects with inherited mutations in selenoprotein biosynthesis with impaired DIO biosynthesis (*SECISBP2* mutations), where TSH was unaltered, whereas the T3 to T4 ratio was reduced and intracellular hypothyroidism was present [37,48,49]. Among the phenotypes of the affected subjects was a delay in bone growth and maturation, which could successfully be corrected for by T3 treatment [50]. Similarly, Se supplementation has proven efficient in preventing or ameliorating symptoms from SELENOP-deficiency, such as growth defects and epileptic seizures, in transgenic mouse models [51]. Considering that the secretory capacity of the thyroid gland (the SPINA-GT index) seemed unaffected in the CFS patients, the suppressed serum T3 to T4 ratio in SELENOP-aAb positive patients is compatible with a defect in local deiodination of TH in Se-sensitive target tissues, like brain, bone, skeletal muscle and others [39,52]. This interpretation is supported by the hypothyroid-like clinical characteristics of CFS, and it is tempting to speculate that SELENOP-aAb lead to an acquired form of resistance to thyroid hormone (A-RTH) with impaired local activation of T4 to T3 [53,54]. Collectively, the physiological consequences from SELENOP-aAb that can be deduced from the data would predict intracellular Se deficiency, low GPx and DIO biosynthesis rate, impaired TH activation in SELENOP-target cells with local hypothyroidism, reduced iodide liberation causing low urinary iodine, and elevated oxidative stress in kidney and elsewhere (Fig. 5).

The interpretation of the data, however, requires additional mechanistic and clinical analyses. To enable such studies, our work identified some useful parameters from body fluids to aid in the detection of Se-related CFS, namely the markers of DIO activity (SPINA GD in combination with the TT3 to TT4 ratio), along with urinary isoprostane and iodine concentrations, collectively covering information on impaired TH deiodination rate and elevated oxidative stress status. Even in case no SELENOP-aAb were detectable in certain patients, the combination of the suggested biomarkers as indicators of (intracellular) selenoprotein deficiency may guide further analyses of alternative causes, such as interfering medication [35,55], Se uptake defects [56,57], (subclinical) chronic inflammation [58,59], malnutrition [[60], [61], [62]], or one of the rare genetic defects impairing selenoprotein expression [63,64].

Among the strengths of this study are a most comprehensive analysis of both the Se and TH status of a set of high-quality samples from CFS patients and their potential interrelationship, providing a consistent picture and a novel plausible pathway for SELENOP-aAb inducing local selenoprotein deficiency, oxidative stress and hypothyroidism in Se-sensitive tissues. Among the notable limitations are the relatively small group sizes and the single time points of analysis, not allowing an analysis of potential longitudinal or causal effects. Moreover, the compelling hypothesis on the iodine excretion shift based on the Dio1-knockout mouse model has not yet completely been tested, as only urine was analyzed. Finally, additional comparisons of clinical parameters and corrections for other potentially relevant sociodemographic factors have not been conducted due to the limited group sizes of controls and patients that were available for analysis. Yet, the findings open a new perspective on this severe disease, provide novel biomarkers of diagnostic and prognostic value, and support a substitution concept as adjuvant personalized therapy.

## 5 Conclusion

A subset of patients with CFS express SELENOP-aAb that are associated with impaired Se transport to target tissues, apparently causing reduced selenoprotein expression and elevated oxidative stress. As local TH activation depends on regular Se supply for DIO expression, SELENOP-aAb may interfere with regular deiodination, impair local T4 to T3 activation, and reduce urinary iodine excretion. SELENOP-aAb positive CFS patients may benefit from supplemental Se intake to bypass the SELENOP-dependent transport route, and T3 substitution to treat local hypothyroidism in Se-dependent tissues, which needs to be tested in clinical studies by applying dosages of Se and T3 that have been proven to be safe and well-tolerated.

## Funding

The research in the lab of LS was supported by the 10.13039/501100001659Deutsche Forschungsgemeinschaft (10.13039/501100001659DFG Research Unit 2558 “TraceAge”, Scho 849/6–2, and 10.13039/501100003383CRC/TR 296 “Local control of TH action”, LocoTact, P17). Research in the lab of EO was supported by a grant from Generalitat valenciana; CIAICO/2021/103. None of the funding sources had any involvement in the study design; in the collection, analysis, and interpretation of data; in the writing of the report; and in the decision to submit the article for publication.

## Author contributions

Qian Sun: Methodology, Validation, Formal analysis, Investigation, Data Curation, Visualization, Writing – Original Draft. Elisa Oltra: Resources, Formal analysis, Investigation, Funding acquisition, Writing – Review & Editing. Janneke Dijck-Brouwer: Resources, Validation, Formal analysis, Writing – Review & Editing. Thilo Samson Chillon: Methodology, Validation, Formal analysis, Investigation, Data Curation, Visualization, Writing – Review & Editing. Petra Seemann: Methodology, Formal analysis, Resources, Investigation, Writing – Review & Editing. Sabrina Asaad: Methodology, Formal analysis, Investigation, Writing – Review & Editing. Kamil Demircan: Methodology, Formal analysis, Investigation, Writing – Review & Editing. José Andrés Espejo-Oltra: Resources, Writing – Review & Editing. Teresa Sánchez-Fito: Resources, Writing – Review & Editing. Eva Martín-Martínez: Resources, Writing – Review & Editing. Waldemar B Minich: Methodology, Formal analysis, Investigation, Writing – Review & Editing. Frits Muskiet: Conceptualization, Resources, Supervision, Writing – Original Draft. Lutz Schomburg: Conceptualization, Resources, Supervision, Funding acquisition, Project administration, Writing – Original Draft.

## Declaration of competing interest

The authors declare that they have no known competing financial interests or personal relationships that could have appeared to influence the work reported in this paper.

LS and PS hold shares of selenOmed GmbH, a company involved in Se status assessment. PS and QS are employees of selenOmed GmbH. LS is listed as inventor on a related patent application. No other relationships or activities are known that could appear to have influenced the submitted work.

## Appendix A Supplementary data

CFS-SELENOP-aAb-RedoxBiol-acc-ls_V3.docx [30] CFS-SELENOP-aAb-RedoxBiol-acc-ls_V3.docx [30]
