# Supplementary material for: Autoantibodies to selenoprotein P in chronic fatigue syndrome suggest selenium transport impairment and acquired resistance to thyroid hormone
Source: Redox Biol. 2023 Jul 3;65:102796. doi: 10.1016/j.redox.2023.102796 (PMC10338150; doi:10.1016/j.redox.2023.102796)
Supplement: CFS-SELENOP-aAb-RedoxBiol-acc-ls_V3.docx [30] [file mmc1.docx]

|  |
| --- |
|  |

**Supplementary Table 1.**

**Anthropometrics of the matched groups of controls and CFS patients from Groningen**

|  | **Control** | **CFS patients** | |  |
| --- | --- | --- | --- | --- |
| **Variable** | n = 119^1^ | **SELENOP aAb(-)***,  n = 99^1^ | **SELENOP aAb(+)***,  n = 12^1^ | p-value^2^ |
| **Age** | 39.0 (25.0) | 42.0 (17.5) | 52.5 (12.8) | 0.052 |
| (Missing) | 30 | 12 | 2 |  |
| **Gender** |  |  |  | 0.6 |
| Female | 76 (77%) | 70 (80%) | 7 (70%) |  |
| Male | 23 (23%) | 17 (20%) | 3 (30%) |  |
| (Missing) | 20 | 12 | 2 |  |
| **BMI** | 22.9 (3.5) | 22.3 (5.7) | 22.8 (2.1) | 0.6 |
| (Missing) | 32 | 12 | 2 |  |
| ^1^Median (IQR) or Frequency (%), ^2^Kruskal-Wallis rank sum test  * threshold for SELENOP-aAb positivity set at BI=5.0 | | | | |
|  | | | | |
